# B-cells and non-B-cells immunoglobulins expression in canine perianal gland tumours: a preliminary study

**DOI:** 10.3389/fvets.2025.1649599

**Published:** 2025-09-17

**Authors:** Claudia Rifici, Elena Angela Lusi, Giada Giambrone, Antonio Ieni, Ettore Napoli, Cornelia Mannarino, Viola Zappone, Giuseppe Mazzullo

**Affiliations:** ^1^Department of Veterinary Sciences, University of Messina, Messina, Italy; ^2^St Vincent Health Care Group-UCD, Dublin, Ireland; ^3^Department of Human Pathology in Adult and Developmental Age "Gaetano Barresi", Section of Pathology, University of Messina, Messina, Italy

**Keywords:** perianal gland tumours, epithelial IgG, B-cells, immunoglobulin, cancer-derived Ig, dog

## Abstract

Although the focus in tumor immunology has been on T cells, B cells may play a crucial role in modulating tumor responses. Among products released in the tumour microenvironment, Immunoglobulins (Igs) have been associated with the development and progression of various types of human cancer. However, their role in veterinary oncology has yet to be fully investigated. It has long been widely acknowledged that Igs are produced solely by B-lineage cells. However, several studies have shown that Ig is also expressed by many normal and pathological “non-B” cells, including neoplastic cells. The aim of this study was to investigate the role of B-cells and Igs in tumours of the canine hepatoglands. Immunohistochemical analysis of ten hepatoid adenomas, thirteen well-differentiated hepatoid carcinomas and nine undifferentiated hepatoid carcinomas revealed that adenomatous structures exhibit the greatest concentration of CD79a-positive, IgA-producing B cells. In contrast, in malignant tumours, an inverse association was observed between CD79a expression and the presence of IgG, accompanied by a significant increase in tumour antigen-specific IgG. These results suggest that IgG produced by neoplastic cells could contribute to tumour progression behavior, potentially serving as prognostic biomarkers and therapeutic targets.

## 1 Introduction

The anal region of dogs is the site of several glandular structures that can undergo neoplastic transformation. These include the anal glands, the pararectal sac glands, and the perianal glands, also known as hepatoid glands. The latter are modified sebaceous glands. They are named for the morphological similarity of their cells to hepatocytes. They are located in the perianal skin, but also in other sites such as the prepuce, the base of the tail, the dorsal region, the groin and the inner surface of the thighs. Their peculiar anatomical distribution, combined with their predisposition to developing neoplastic lesions, makes them clinical and pathological interest.

Hepatoid gland neoplasms (HGNs) are a notable health issue in male dogs, representing 9–18% of skin cancer cases and ranking third in tumor incidence after other cutaneous tumors and testicular tumor (1, 2). Hepatoid gland adenomas and differentiated adenocarcinomas, are significantly influenced by androgens and estrogens, as evidenced by the presence of steroid hormone receptors and the observed neoplastic regression following castration. These tumors have a higher incidence in intact male dogs, particularly those over 10 years of age, indicating a strong link between sexual hormones and tumor development (3–5).

The prognosis varies based on histological features and clinical stage. Benign tumours such as adenomas generally show an excellent prognosis after surgical excision. In contrast, malignant hepatoid carcinomas display variable outcomes depending on invasiveness and tumour size, with smaller, minimally invasive carcinomas often associated with a more favorable prognosis than larger ones (1).

Macroscopically, HGNs are characterized by single or multiple often ulcerated masses surrounding the anus, tail, hind limbs, parapreputial and vulvar areas (6). In particular, well-differentiated carcinomas are clinically indistinguishable from adenomas, as both manifest as nodular formations. In contrast, poorly differentiated carcinomas frequently show ulceration and have poorly defined borders (7).

Histologically, they are classified into three main types: benign hepatoid gland adenomas (HGAs), potentially malignant hepatoid gland epitheliomas (HGEs) and hepatoid gland carcinomas (HGCs), each with distinct features (6, 8).

Adenomas show well-organized cell islands with small, hyperchromatic nuclei and eosinophilic cytoplasm, while epitheliomas form lobules with basaloid cells exhibiting larger nuclei, prominent nucleoli, and occasional mitoses, sometimes showing squamous metaplasia or anaplasia (9). Carcinomas, especially well-differentiated types, retain hepatoid morphology, forming solid islands and trabeculae with a cribriform pattern, whereas undifferentiated carcinomas display more aggressive, poorly organized structures (8, 9). These histopathological differences influence diagnosis and treatment.

HGAs are more diffuse commonly observed (58–96%) than HGCs (3–21%). HGCs grow rapidly and can metastasize to regional lymph nodes, however distant metastases to abdominal organs and lungs are rare (1, 10). Despite HGNs relatively high incidence, a clear etiology of hepatoid gland tumours has not yet been established (4). Current research reveals significant gaps in knowledge regarding the immune response and immunoglobulin expression in these tumors, hindered by a lack of studies addressing the interaction between immune cells, tumor characteristics, and stromal infiltration. Notably, the role of immunoglobulins expressed by B cells and non-B cells, including cancer cells, has not been thoroughly investigated in the development and progression phatobiology of these tumors.

Immunoglobulins (Igs), while traditionally associated with B cells and immune regulation, are also expressed by a variety of normal and pathological “non-B-cells,” including podocytes (11), gametes (12), neurons (13), epithelial cells (14, 15), endothelial cells (16) glandular cells (17) and even neoplastic cells may express immunoglobulins (18). This expansion of Ig expression beyond B cells underscores the complex roles that Igs may play in both normal physiological processes and various pathological conditions. Non-B cell derived immunoglobulins (Igs) play a dual role in biology; under normal physiological conditions, they contribute to immune defense, but during malignant transformation, they can promote tumorigenesis. Research indicates that these non-B Igs facilitate tumor growth (19), enhance cell migration and invasion (20) and improve cell adhesion, highlighting their complex involvement in cancer progression (11, 21).

This pioneering work sheds light on the often-overlooked role of B cells (BC), plasmacell and immunoglobulins in tumor immunology by analyzing the differences in immunoglobulin expression between B cells and non-B cell epithelial cancer cells in canine hepatoid gland tumors, emphasizing the relationship between immune cell profiles and tumorhistological classification. By examining the mechanisms of non-B immunoglobulin production and regulation, the research seeks to provide deeper insights into the immune landscape of these tumors, which could inform more effective diagnostic and therapeutic strategies in veterinary oncology, ultimately enhancing our understanding of canine malignancies.

## 2 Materials and methods

### 2.1 Animals, tissue processing, and histopathology

Formalin-fixed and paraffin-embedded tissue blocks of 32 canine hepatic gland tumors were retrieved from the archives of the Unit of Veterinary Pathology, Department of Veterinary Sciences, University of Messina. Sections were cut from paraffin blocks (5 μm) and stained with hematoxylin and eosin (HE) for histopathological assessment. Slides were reviewed by two veterinary pathologists (CR andGM).

The classification of hepatoid gland tumors was based on criteria proposed by Goldschmidt and Goldschmidt (8) and we further specified the morphological features used to differentiate adenomas, epitheliomas, well-differentiated and poorly differentiated carcinomas, including cellular architecture, nuclear atypia, mitotic index, and evidence of invasion (9). The analysed cohort consisted of: 10 cases of hepatoid adenoma, 13 cases of differentiated carcinoma and 9 cases of undifferentiated carcinoma. Of the 10 perianal gland adenomas, 9 cases were male, and one were female, aged 6 and15 years. The adenomas were seen in Siberian Husky (*n =* 1), Poodle (*n =* 1), Shi-Tzu (*n =* 1), English bulldog (*n =* 1), Spinone (*n =* 1), Yorkshire Terrier (*n =* 1), German Shepherd (*n =* 1), Maltese (*n =* 1) and crossbred (*n =* 2). The well-differentiated carcinomas were diagnosed in 12 males and 1 female, aged 6–15 years, of the following breeds: West Highland White Terrier (*n =* 2), Fox Terrier (*n =* 1), Irish Setter (*n =* 1), English Setter (*n =* 1), Poodle (*n =* 1), Dalmatian (*n =* 1), Husky (*n =* 1), and crossbred (*n =* 5). All undifferentiated carcinomas occurred in male dogs aged 10–14 years, including Fox Terrier (*n =* 1), Bull Terrier (*n =* 1), Husky (*n =* 4), and crossbred (*n =* 3).

The phenotypic characterization of B-cell (BC) was performed by methyl green pyronine (MGP), a histochemical stainthat highlights RNA-rich plasma cells and actively transcribing B lymphocytes, and immunohistochemistry (IHC) for CD79a. The use of MGP allowed better identification of B-cell populations in combination with IHC. Slides of each sample underwent direct immunofluorescence (IF) for immunoglobulins and indirect immunofluorescence for CD79a (the primary Abs are detailed in Table 1).

**Table 1:** Primary antibodies used for IHC and IF.

Antibody	code/clone	Brand	Specificity	Dilution
CD79a	HM47-A9	Novocastra HM47-A9	B- Cell receptor	1:50
Sheep Anti Dog IgG: FITC	n/a	AbD Serotec	Immunoglobulin G	1:100
Mouse Anti Dog IgG: FITC	n/a	Sigma	Immunoglobulin G	1:100
Goat Anti Dog IgA: FITC	n/a	AbD Serotec	Immunoglobulin A	1:100

### 2.2 Immunohistochemistry and immunofluorescence

Five micrometer slices were steamed in 0.01 mol/L sodium citrate buffer, pH 6, in a microwave oven for 15 min. Endogenous peroxidase activity was quenched by 0.3% hydrogen peroxide in methanol, while aspecific protein reactions were blocked by incubation with 2.5% BSA for 30 min.

For immunofluorescence, an additional bath of 10 min in 0.1% sodium borohydride (Merck, KGaA, Darmstadt, Germany), to reduce autofluorescence, was performed. Slides were after incubated over night at 4 °C with primary FITC conjugated Abs followed by rinsing in PBS and cover slipping for IF (Vectashield anti fade mounting medium with dapi, Vector Laboratories, Inc., Burlingame, CA, USA) or an incubation at room temperature with a biotinylated goat anti-mouse (BIOSPA, SPA Società Prodotti Antibiotici, Milan, Italy) or peroxidase conjugated anti-mouse (Santa Cruz Biotechnology, Dallas, TX, USA) secondary Ab for IHC or TR conjugated goat anti-mouse secondary Ab for IF (Santa Cruz Biotechnology, Dallas, TX, USA).

An additional reaction was carried out by an avidin-peroxidase complex for biotinylated secondary antibody (BIOSPA, SPA Società Prodotti Antibiotici, Milan, Italy). All the immunohistochemical reactions were developed with Vector Nova Red (Vector Laboratories, Inc., Burlingame, CA, USA) and counterstained with hematoxylin. For each sample negative control was also performed by omission of primary Ab or substitution with normal immunoglobulins from the same species of primary Abs. Positive controls were represented by canine lymph node sections (for CD79a) and canine tonsil tissue (for IgA/IgG), which consistently showed the expected reactivity Immunohistochemical stains were interpreted by assessing the cytoplasmic and/or membrane immunoreactivity. The slides, prepared as above, were observed under an optical microscope (DMI6000, Leica Microsystems) connected to a camera and image analysis software (Leica Application Suite X, Leica Microsystems), and a numerical count of stained immune cells was performed. In the neoplastic lesions 10 intratumoral and 10 peritumoral microscopic fields were observed at 40x magnification and the numerical count of BC was performed. Each field corresponded to ~0.0625 mm^2^ (250 × 250 μm). Fields were randomly selected. The same evaluation method was applied to IgA and IgG IF slides as well as to methyl green pyronine histochemistry.

### 2.3 Statistical analysis

The analysis of B-cells in intratumoral and peritumoral regions, as well as among different malignant histotypes, was conducted using the Kolmogorov–Smirnov test for normality, followed by Student’s *t*-test to assess differences between groups and one-way ANOVA for comparisons across malignant types. Correlation analyses between marker expression and tumour type were assessed using Spearman’s correlation test, reporting coefficients (r) and *p*-values where significant.

Data processing was performed using Microsoft Excel for Mac (16.44), while statistical analysis was executed with GraphPad Prism version 5.1, with significance set at a *p*-value of less than 0.05.

## 3 Results

**Figure 1:**
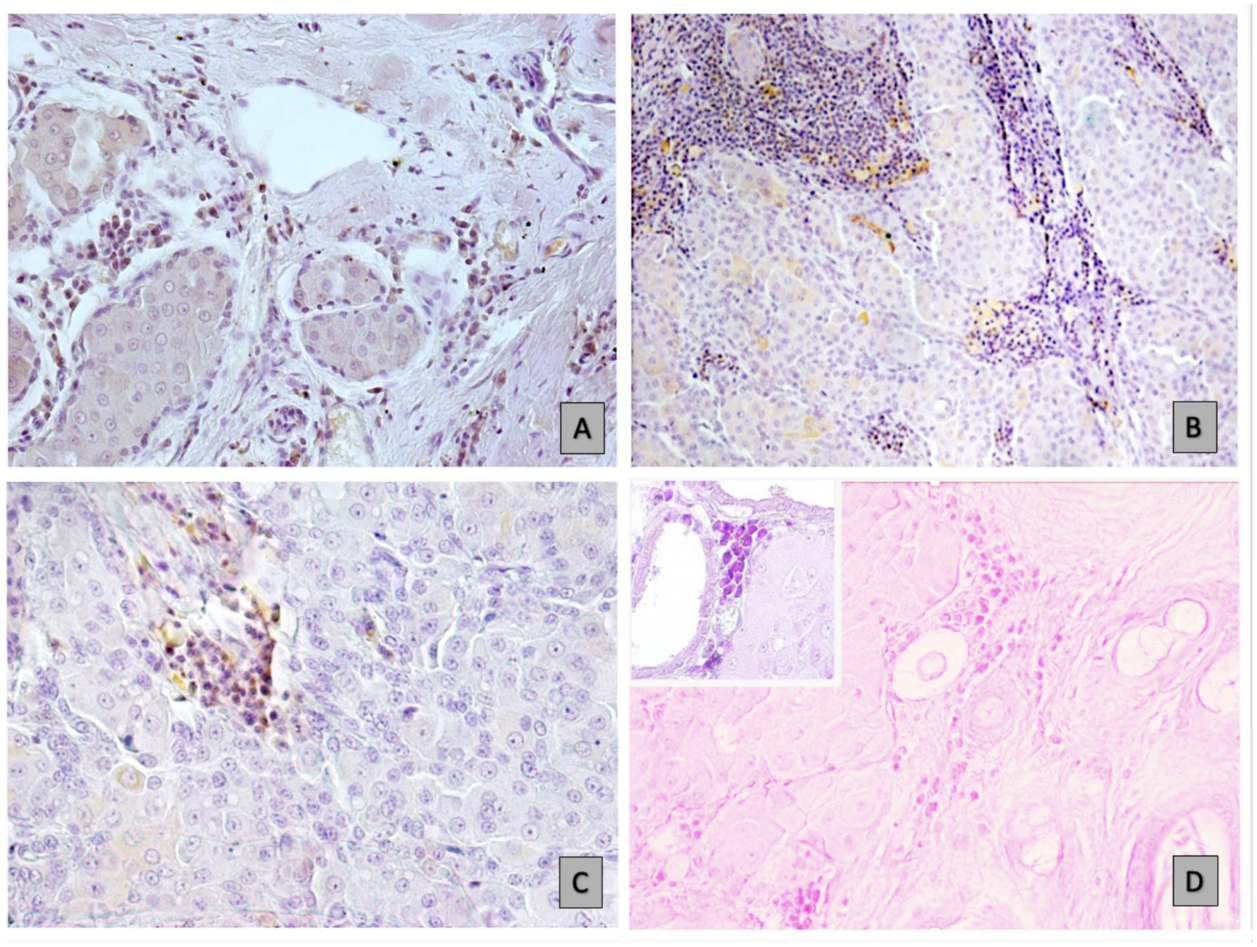
B-cell and plasma cell distribution in different tumour environments: immunohistochemical (IHC) analyses. **(A)** Infiltration in hepatoid carcinoma, as indicated by CD79a staining at 20x magnification. **(B)** High numbers of immune cells in the peristroma of carcinoma, stained for CD79a at 10x magnification. **(C)** Sparse in well-differentiated carcinoma areas (20x magnification). **(D**) Minimal B-cell and plasmacell presence in undifferentiated carcinoma (10x magnification), as well as at a higher magnification of 40x (insert), using MGP.

The immunohistochemical analyses revealed the presence of CD79-positive B cells and plasma cell in various locations within the tumor microenvironments. CD79-positive B cells and plasma cell infiltrate the peritumoral stroma of adenoma (*p* < 0.0001) (Figure 1A), and prominently the peritumoral regions surrounding well-differentiated carcinomas (p < 0.0001) and undifferentiated carcinomas (*p* = 0.0430) (Figure 1B) compared to the intratumoral stroma. Instead, in the intratumoral stroma, the observation revealed a numerical decline in B cell and plasma cell in differentiated carcinoma regions and minimal presence in undifferentiated aggressive cancer lesions (*p* = 0.00001), as shown in Figures 1C,D. The mean and standard deviation of B cell counts in peritumoral and intratumoral regions across three types of tumors: adenoma, well differentiated carcinoma, and undifferentiated carcinoma are illustrated in Table 2. Well-differentiated carcinomas showed lower intratumoral counts than adenomas, and the lowest values were observed in undifferentiated carcinomas.

**Table 2:** Mean and standard deviation (SD) of B cell counts in peritumoral and intratumoral regions across three types of hepatoid glands tumors:

Type of tumour	B cells Peri-tumoral		B cells Intra-tumoral	
	Mean	SD	Mean	SD
Adenoma	34,49	6,77,388	13,16	4,553,192
Well differentiated Carcinoma	62,825	14,44,485	1,2	0,417,475
Undifferentiated Carcinoma	65,84,286	13,65,123	1,228,571	0,40,708

**Figure 2:**
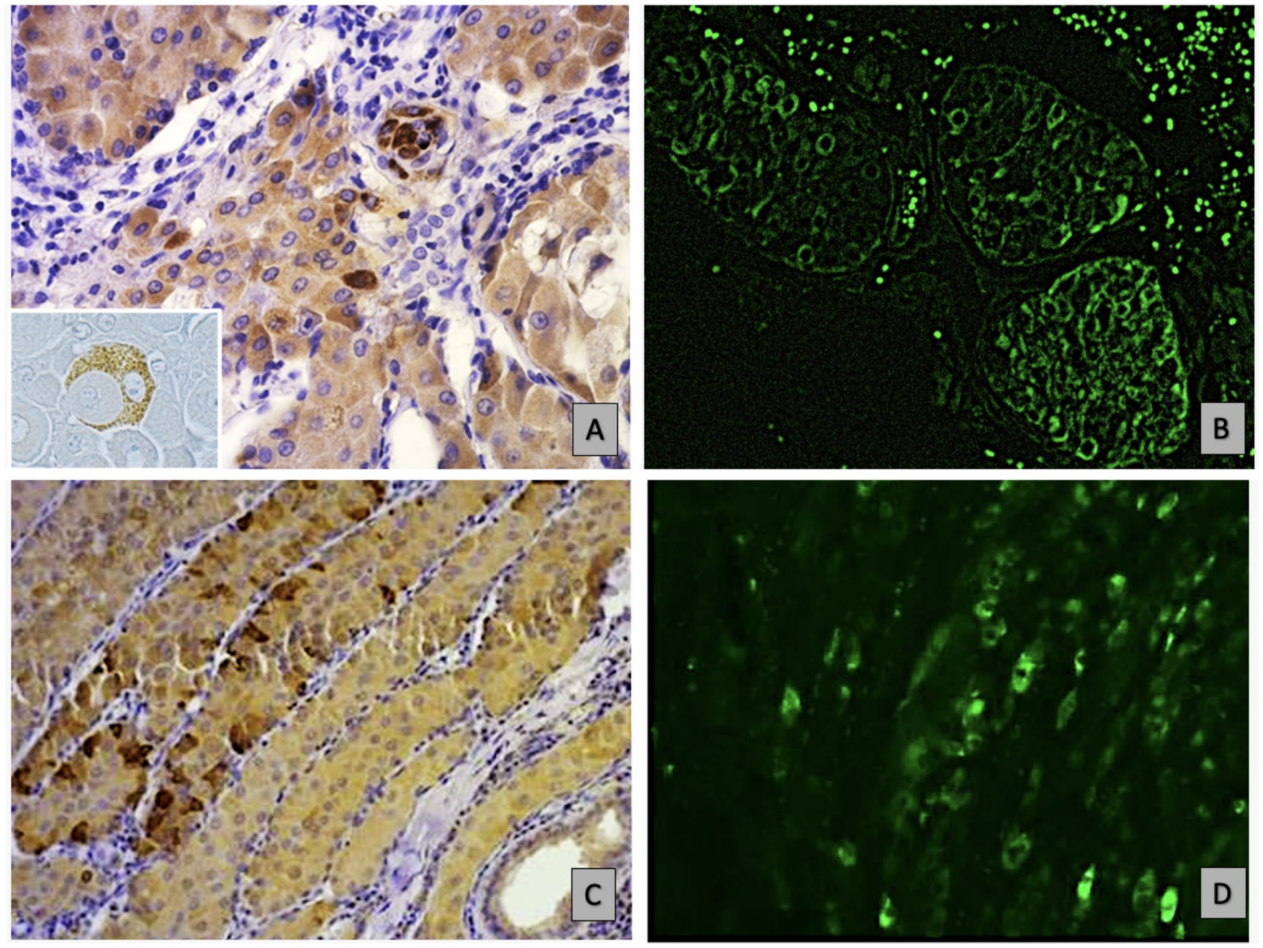
Shows immunohistochemical (IHC) and immunofluorescence (IF) analyses of non-B hepatoid cancer epithelial cells, highlighting granular hepatoid cells in an adenoma: **(A)** IHC staining for CD79a at 40x, revealing granular CD79 positivity in epithelial cells, as highlighted in the insert. **(B)** Depicts homogeneous IgA expression in these cells via IF at 10x. **(C)** Identifies well-differentiated carcinoma cells at the lobule periphery through IHC for CD79a at 20x, while D) shows a similar distribution of IgA expression using IF at the same magnification.

**Figure 3:**
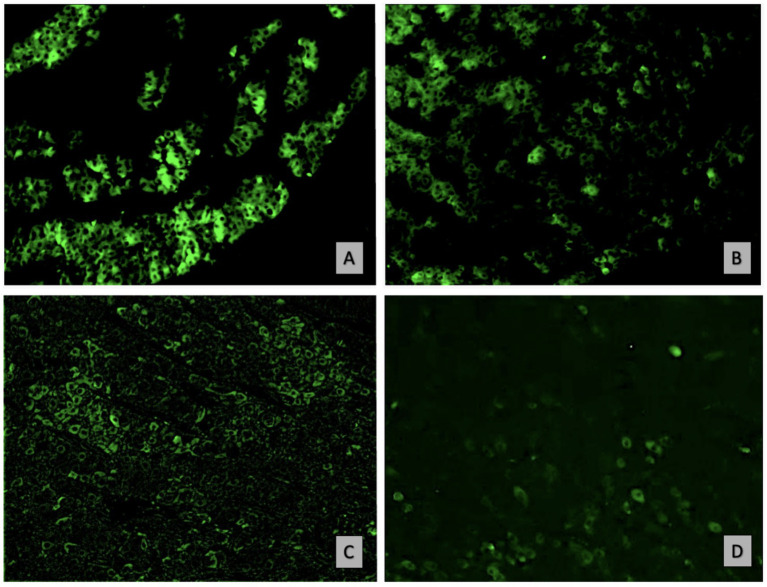
**(A)** Intensely IgG-positive neoplastic hepatoid cells in well-differentiated carcinoma (IF for IgG-FITC, 20x) and **(B)** in undifferentiated carcinoma (IF for IgG-FITC, 20x); **(C)** Reduced IgA positivity in malignant histotypes (IF for IgA-FITC, 20x). **(D)** Rare cells positive for the CD79a marker in undifferentiated carcinoma (IF for CD79a, 20x).

The observation indicates an atypical expression of CD79a in epithelial neoplastic cells showing a distinct granular positivity and dispersed cytoplasmic distribution, as illustrated in Figure 2A. The granular staining pattern may reflect either aberrant protein expression by neoplastic cells or, alternatively, a non-specific signal. Further studies are required to clarify this aspect Epithelial neoplastic cells in adenomas also strongly expressed IgA, with the CD79a and IgA positive cells primarily localized in the central lobules or occasionally found at the periphery, Figures 2A,B. In contrast, carcinoma lesions show a reduction in both B cells and non-B cells that express CD79 (Figure 2C). The expression of IgA was reduced in undifferentiated carcinomas compared to adenomas and well-differentiated carcinomas. (Figure 2D). In malignant tumours, the immune response was characterized by fewer B cells, alongside a greater proportion of epithelial cells that had downregulated CD79 expression and IgA production, correlating with significantly higher IgG levels. The positivity of the two tested IgG antibodies was consistent in both presence and intensity. This highlightis a shift difference in immune dynamics across tumor types (Figures 3A–D).

### 3.1 Results summary

Histochemical and immunohistochemical analyses of canine hepatoid gland tumours revealed that B cells, particularly those expressing CD79a, were significantly higher in adenomas and in the surrounding stroma of well-differentiated carcinomas, suggesting their protective role. In adenomas, non-B cancer cells also demonstrate CD79 positivity and produced IgA, suggesting some interaction with B cell activity. This co-expression was observed by comparing serial sections but does not prove direct interaction between epithelial cells and B cells.

Epithelial positivity for IgA closely aligned with that of CD79a in both benign and malignant lesions. However, undifferentiated carcinomas showed fewer B cells and lower IgA expression, alongside significantly higher IgG positivity by cancer cells.

This may reflect immune evasion and a more aggressive tumour phenotype. Immunohistochemistry results support the notion that cancer-derived IgG may influence tumour behavior, by enhancing invasion and metastasis.

However, in this study the association between IgG expression and metastatic spread could not be assessed for due to a lack of clinical data.

## 4 Discussion

The debate surrounding canine hepatoid glands highlights the uncertainty regarding their physiological role, with conflicting views on whether they act as odoriferous exocrine glands or serve a role in steroid metabolism. This ambiguity extends further into their pathological potential, as current research is insufficient in exploring the tumor microenvironment of these glands.

Compared to T lymphocytes, there have been comparatively few research on the function of B lymphocytes and immunoglobulins in cancer microenvironments, both in human and animal tumors. B cells, traditionally known for their role in antibody production and immune response, have increasingly been recognized for their complex involvement in cancer biology. While T cells have dominated research narratives in cancer immunity (22), in tumor tissues, B lymphocytes account for a high proportion of cells and are composed of activated, antigen-presenting, and memory B-cell subpopulations. These cells produce antibodies that directly target tumors, promote tumor recognition and tumor cell clearance through the activation of macrophages and complement cascades and antibody-mediated cytotoxicity, and play an important role in tumor antigen identification (23). However, sometimes B cells have a promoting effect on tumors, which is usually associated with the presence of immunosuppressive B-cell subsets called “regulatory B cells,” which may support tumor progression through various mechanisms. The existence of this antagonistic effect is due to the influence of different internal tumor environments anatomical regions and local microenvironments, which cause the differentiation of B cells into different functional phenotypes and result in divergent immune outcomes.

In fact, while studies have shown that B-cell abundance in cancer is positively associated with favorable clinical outcomes, others have indicated that they are tumour-promoting, implying that the biological function of B cells is a complex landscape. B cells can promote tumorigenesis through various mechanisms, including the modulation of immune responses and the upregulation of pro-angiogenic genes. In both murine models and human subjects, B cells have been shown to support carcino-genesis by producing antibodies that form immune complexes within tumor tissues, thereby exacerbating inflammation and facilitating neoplastic progression (24).

Recent studies in veterinary oncology have begun to explore the presence and role of B cell infiltration in the tumor microenvironment, revealing that CD79a and CD20 positive B cells are found in feline oral squamous cell carcinoma (25) and canine mammary tumors (26). Additionally, research by Perez et al. (27) highlighted the dynamics of B cell populations in Canine Transmissible Venereal Tumor (CTVT), indicating a potential link between B cell response and tumor behavior, where specific immunoglobulin profiles could reflect the tumor’s progression or regression. However, these studies primarily focused on classical B-cell-derived immunoglobulins, and little is known about the role of non-B-cell-derived Ig in veterinary cancers. In our pilot study, we used CD79a to assess B-cell infiltration in canine hepatoid gland tumours. CD79a is a B-cell receptor component, essential for B-cell activation, proliferation, and differentiation. CD79 is a heterodimeric protein, comprised of two transmembrane subunits CD79a and CD79b, which are ex-pressed mainly on B cells and serve as a critical component of the B-cell receptor complex. It plays an essential role in B cell activation, proliferation, and differentiation. Due to its specificity for B cells, CD79a is commonly used as a marker in immuno-histochemistry to identify and study B cell-related disorders, such as lymphomas and leukemias (28).

Our study therefore enriches the landscape of other canine tumours has also been investigated, e.g., mast cell tumours, melanomas, and soft tissue sarcomas, showing prognostic roles for tumour-infiltrating lymphocytes and macrophages 22, 29, 30. Expanding this comparative view would strengthen future analyses. Unexpectedly, we also found CD79a positivity in epithelial cells. This atypical expression may suggest a potential role of CD79a in the pathobiology of these neoplastic cells.

Interestingly, while studying the presence of B cells using CD79 as a marker, we found an unexpected expression of CD79 in epithelial cells, aside from its typical presence in B cells. The unexpected presence of CD79 in epithelial cells is an intriguing finding that suggests that some markers traditionally associated with B cells may play roles in non-hematopoietic tissues as well.

This atypical expression is intriguing but requires further validation. It may reflect either genuine ectopic expression or an artefact. Confirmatory analyses (e.g., Western blot, RNA scope, sequencing) are necessary before concluding that neoplastic epithelial cells express CD79a. In addition, we observed Igs expression from epithelial cells, which is consistent with earlier findings that immunoglobulins, which are typically associated with B cells and their traditional roles in antibody-mediated immunity, may also be present in a variety of bodily tissues, including epithelial cells.

According to classical immune theory, B lymphocytes have been considered the only source of Ig production (B-Igs). However, emerging research indicates that non-B cells, particularly those found in various human epithelial tumors, can produce immunoglobulins (Igs), challenging the classical understanding that B lymphocytes are the sole source of Ig production. These non-B Igs may exhibit distinct structural differences from B-cell-derived Igs, which could result in varied functions that influence cancer biology (31).

Regarding the dualism of B lymphocytes in cancer, their “friend” role was particularly evident in our observations of hepatoid gland tumor microenvironments. The significant presence of B cells in the peritumoral stroma around cancer lesions, attempting to prevent the disease’s invasion, suggests that B cells may play an important, although frequently underestimated, role in the immune response to cancer.

This finding shifts the focus from the traditionally emphasized T cell-mediated immunity to highlight the potential importance of B cells in containing tumor spread and contributing to antitumor immunity.

As previously observed in canine mammary tumors (32), the unexpected finding of CD79 and IgA expression in epithelial neoplastic cells might reflect complex cross-talk between neoplastic and immune cells. However, co-localisation studies (e.g., double staining) would be required to confirm a direct relationship, which we did not perform in this pilot study This expression pattern, with epithelial cells exhibiting positivity for both CD79a and IgA, may indicate a shared or overlapping pathway in immune response or tumor immunology, warranting further investigation to elucidate the mechanisms underlying this phenomenon and its implications for tumor behavior and treatment.

Our data show that malignant tumours exhibit fewer B cells and lower IgA, while IgG expression is higher. This difference may indicate immune evasion. However, we did not observe metastases in our cases, and thus cannot link IgG to metastatic potential here. In contrast, adenomas demonstrate a wealth of B cells producing diverse immunoglobulins, indicating a more robust immune response. Tumor-derived IgG appears to enhance the survival and proliferation of epithelial tumor cells through autocrine or paracrine mechanisms, potentially allowing tumors to evade immune responses by competing with B cell-derived antibodies for Fc receptors on effector cells, thereby inhibiting antibody-dependent cellular cytotoxicity (ADCC). Evidence suggests that poorly differentiated carcinomas express higher levels of IgG, while B-cell infiltration and antibody production may precede tumor development and promote growth (20). The idea that tumour-derived IgG supports tumour growth and immune evasion has been proposed in human oncology (19, 20, 33–36). In our context, it remains a hypothesis needing validation.

Taken together, our study highlights that both B cells and possible non-B-cell Ig expression may contribute to the hepatoid tumour microenvironment. But their exact role (protective vs. tumour-promoting) remains unclear, and additional validation is necessary.

## 5 Conclusion

Canine hepatoid gland tumors present a pivotal opportunity for advancing research in translational oncology by examining the complex role of immunoglobulin production in non-hematopoietic tumors. For example, comparing immunoglobulin patterns in hepatoid gland tumors and Canine Transmissible Venereal Tumor (CTVT) could reveal similarities in immune response mechanisms given their anatomical proximity in the reproductive region. Such comparative studies may highlight shared pathways in tumor development and regression.

Our preliminary findings indicate differences in B-cell infiltration and immunoglobulin expression between benign and malignant lesions. However, interpretation must be cautious: many hepatoid adenomas remain benign, and our data do not demonstrate a mandatory progression towards carcinoma. Furthermore, the atypical expression of CD79a and IgA in epithelial cells should not yet be considered proof of immunoglobulin synthesis by neoplastic cells. Confirmatory analyses (e.g., Western blot, RNA scope, sequencing) are required before drawing such conclusions.

This study is limited by its small sample size and preliminary nature. Nevertheless, it highlights the importance of further investigations into the dual functions of immunoglobulins, the role of tumour-derived IgG, and the potential of B cells as both biomarkers and therapeutic targets in veterinary oncology. By extending this knowledge, veterinary medicine could contribute to more personalised treatment strategies and, through comparative oncology, provide insights also relevant to human cancers.

## Data Availability

The original contributions presented in the study are included in the article/supplementary material, further inquiries can be directed to the corresponding authors.
